# Identification of Enhancers and Promoters in the Genome by Multidimensional Scaling

**DOI:** 10.3390/genes12111671

**Published:** 2021-10-23

**Authors:** Ryo Ishibashi, Y-h. Taguchi

**Affiliations:** 1 Graduate School of Science and Engineering, Chuo University, Tokyo 112-8551, Japan; 2 Department of Physics, Chuo University, Tokyo 112-8551, Japan; tag@granular.com

**Keywords:** multidimensional scaling, high-throughput chromosome conformation capture, enhancer, promoter

## Abstract

The positions of enhancers and promoters on genomic DNA remain poorly understood. Chromosomes cannot be observed during the cell division cycle because the genome forms a chromatin structure and spreads within the nucleus. However, high-throughput chromosome conformation capture (Hi-C) measures the physical interactions of genomes. In previous studies, DNA extrusion loops were directly derived from Hi-C heat maps. Multidimensional Scaling (MDS) is used in this assessment to more precisely locate enhancers and promoters. MDS is a multivariate analysis method that reproduces the original coordinates from the distance matrix between elements. We used Hi-C data of cultured osteosarcoma cells and applied MDS as the distance matrix of the genome. In addition, we selected columns 2 and 3 of the orthogonal matrix U as the desired structure. Overall, the DNA loops from the reconstructed genome structure contained bioprocesses involved in transcription, such as the pre-transcriptional initiation complex and RNA polymerase II initiation complex, and transcription factors involved in cancer, such as Foxm1 and CREB3. Therefore, our results are consistent with the biological findings. Our method is suitable for identifying enhancers and promoters in the genome.

## 1. Introduction

For cells to utilize genetic information, many genes must be expressed in a coordinated manner. The accessibility of genomic information depends on how DNA is packed into the chromatin. Chromatin is the basis of various biological processes, including cell cycle regulation and, DNA replication, repair, and maintenance [1]. Euchromatin is a genome region consisting of DNA with a relatively loose structure. The open structure allows RNA polymerase and other proteins to access the genome for DNA transcription. Enhancers and promoters also approach the euchromatin region to form DNA loops. Gene expression is controlled by promoters near the gene as well as by gene regulatory sites named as enhancers that are distant from the gene. However, how promoters and enhancers interact with each other to regulate gene expression is not well understood. High-throughput chromosome conformation capture (Hi-C) can be used to analyze the 3D structure of a genome by detecting genomic regions that are spatially close to each other using next-generation sequencing [2]. This conventional method led to an approximation of the genome structure from the Hi-C heat map [3]. We demonstrated the potential of using this method for identifying enhancers and promoters by applying multi-dimensional scaling (MDS).

## 2. Materials and Methods

### 2.1. Hi-C Data

**Figure 1:**
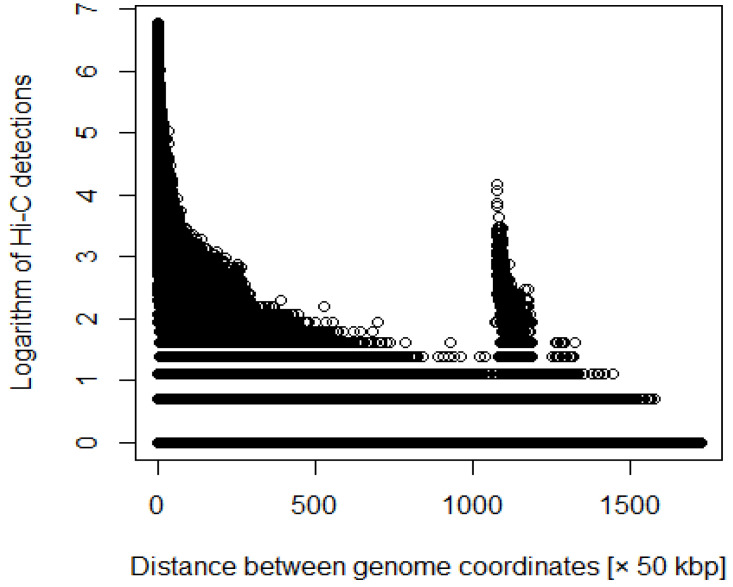
Distance between coordinates versus logarithm of Hi-C detections before adding weighting.

**Figure 2:**
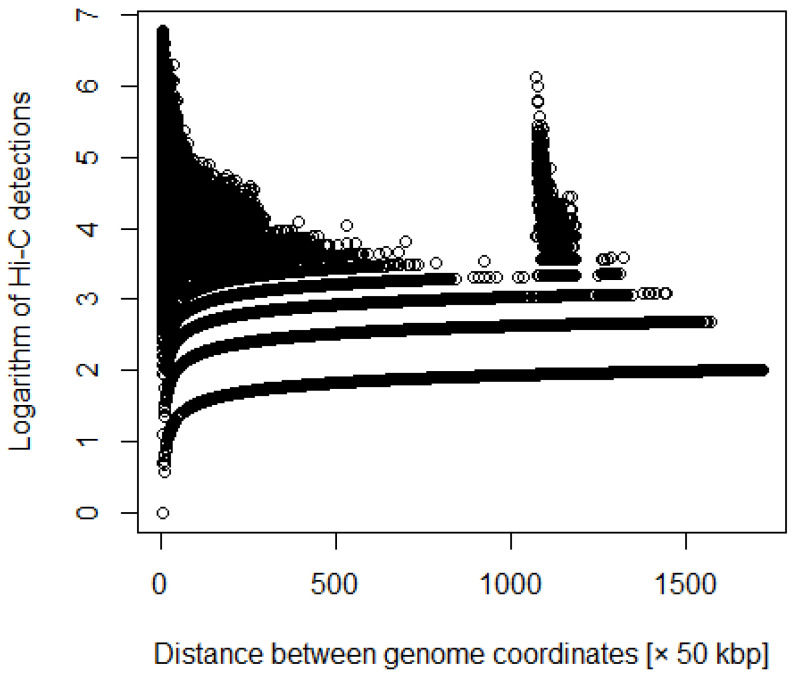
Distance between coordinates versus logarithm of Hi-C detections after adding weighting.

The Hi-C dataset is valuable for understanding how chromatin is organized in the nucleus to effectively perform its biological functions; that is, it enables examination of the physical interactions of DNA loops. We downloaded eight Hi-C data during the cell cycle: 0 min (metaphase) and 35 min (anaphase/telophase); 60 min (cytokinesis); and 90, 120, 180, 240, and 360 min (G1) from Series GSE141067 [2]. In this study, we analyzed Hi-C data with a resolution of 50 kbp and found that long-range genomic interactions were reshaped from 60 min and completed within 90–120 min [2]. We assigned the average number of Hi-C detections for each distance between genomic coordinates to the missing values. In addition, the Hi-C data showed a significant difference between the number of detections with a far distance between coordinates and the number of detections with a close distance. Equation (1) is a function that does not affect the number of detections when the distance between the coordinates is close but increases the number of detections when the distance between the coordinates is large. As the purpose of this study was to identify enhancers and promoters, we evaluated those with a large number of Hi-C detections, although the distance between coordinates was large. Therefore, the following steps were performed according to the distance between nucleotides (Supplementary Tables S1–S4).

(1) $$d_{ij}\mathrm{new}=\left\{\begin{array}{c} d_{ij}\;\;,|i-j|\leq 5\;\\ d_{ij}\times \log|i-j|\;,\;|i-j|>5\; \end{array}\right.$$

where *i* and *j* are coordinates, and *d*${}_{ij}$ is the number of Hi-C detections (Figure 1 and Figure 2). We processed the Hi-C data with various weights. For example, we multiplied the number of detections by the distance between each coordinate.

(2) $$d_{ij}\mathrm{new}=\left\{\begin{array}{c} d_{ij}\;\;,|i-j|\leq 5\;\\ d_{ij}\times a|i-j|\;,\;|i-j|>5\; \end{array}\right.$$

where *a* is a constant. We then averaged the number of detections per coordinate distance and multiplied the number of detections greater than the average by a constant.

(3) $$d_{ij}\mathrm{new}=\left\{\begin{array}{c} d_{ij}\;\;,d_{ii+j}\leq \frac{\Sigma_{i=0}^{N-j}d_{ii+j}}{N-j},\left(j=0,1,\ldots ,N\right)\;\\ d_{ij}\times a\;,\;d_{ii+j}>\frac{\Sigma_{i=0}^{N-j}d_{ii+j}}{N-j},\left(j=0,1,\ldots ,N\right)\; \end{array}\right.$$

where *N* is the length of the base. However, the results of enrichment analysis obtained using methods (2) and (3) were unsatisfactory.

**Figure 3:**
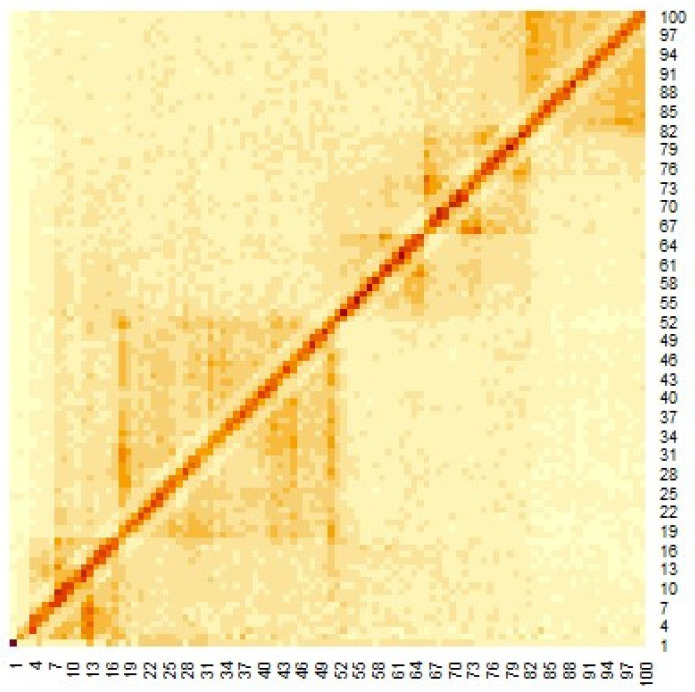
Heat map of Hi-C data after adding weighting.

Figure 3 shows the heat map of the Hi-C data, where the genomes are close to each other when the number of Hi-C detections was large. To apply the multidimensional scale construction method, the Hi-C data were transformed as follows:

(4) $$D_{ij}=\frac{1}{d_{ij}\mathrm{new}}$$

The inverse of the Hi-C data was used as the distance data because the multidimensional scale construction method was used for similarity matrices.

### 2.2. Multidimensional Scaling

MDS (Appendix A) is a method that reproduces the original location of objects based on the distance data between objects [4] according to the following principle. Consider an N×P data matrix $X=(x_{ij})$, *N* data $o_{i}=\left(x_{i1},x_{i2},....,x_{iP}\right)$. Then, we consider the N×N matrix $B={\mathrm{XX}}^{t}$ and the N×N distance matrix $D=(||o_{i}-o_{j}||)$ created from X. We define $D^{\left(2\right)}$ as the matrix of all components of the distance matrix D squared, and multiply $D^{\left(2\right)}$ by the N×N centralization matrix $J(=E-\frac{1}{N}1)$ from both sides.

(5) $$\begin{array}{cc} -\frac{1}{2}JD^{\left(2\right)}J^{t} & =-\frac{1}{2}J\left\{\mathrm{diag}\left({\mathrm{XX}}^{t}\right)1-2{\mathrm{XX}}^{t}+1\mathrm{diag}\left({\mathrm{XX}}^{t}\right)\right\}J^{t}\\ \; & =\mathrm{JX}X^{t}X^{t}=\mathrm{JX}{\left(\mathrm{JX}\right)}^{t}\\ \; & =X^{*}X^{*t}=B_{cen} \end{array}$$

$X^{*}$ is the centered data matrix and $B_{cen}$ is the inner product matrix obtained from the centered data matrix.

(6) $$G^{t}B_{cen}G=\left(\begin{array}{ccccccc} \lambda_{1} & \; & & \; & \\ \; & \lambda_{2} & \; & & 0 & \\ \; & \; & \ddots & \; & \\ \; & \; & & \lambda_{P}\\ \; & \; & & \; & 0\\ \; & 0 & \; & & \; & \ddots \\ \; & \; & & \; & & \; & 0 \end{array}\right)$$

(7) $$\begin{array}{cc} B_{cen} & =G^{t}\left(\begin{array}{ccccccc} \sqrt{\lambda_{1}} & \; & & \; & \\ \; & \sqrt{\lambda_{2}} & \; & & 0 & \\ \; & \; & \ddots & \; & \\ \; & \; & & \sqrt{\lambda_{P}}\\ \; & \; & & \; & 0\\ \; & 0 & \; & & \; & \ddots \\ \; & \; & & \; & & \; & 0 \end{array}\right)\\ \; & \times \left(\begin{array}{ccccccc} \sqrt{\lambda_{1}} & \; & & \; & \\ \; & \sqrt{\lambda_{2}} & \; & & 0 & \\ \; & \; & \ddots & \; & \\ \; & \; & & \sqrt{\lambda_{P}}\\ \; & \; & & \; & 0\\ \; & 0 & \; & & \; & \ddots \\ \; & \; & & \; & & \; & 0 \end{array}\right)G \end{array}$$

G is the orthogonal matrix of the inner matrix $B_{cen}$. From (5) and (7),

(8) $$X^{*}=G^{t}\left(\begin{array}{ccccccc} \sqrt{\lambda_{1}} & \; & & \; & \\ \; & \sqrt{\lambda_{2}} & \; & & 0 & \\ \; & \; & \ddots & \; & \\ \; & \; & & \sqrt{\lambda_{P}}\\ \; & \; & & \; & 0\\ \; & 0 & \; & & \; & \ddots \\ \; & \; & & \; & & \; & 0 \end{array}\right)$$

From the above equations, the original coordinates can be derived.

### 2.3. Applying MDS to the Hi-C Data

We applied MDS to the distance matrix *D*

(9) $$D=ULU^{t}$$

It is unclear which column of the orthogonal matrix U presents the desired structure. However, in many cases, it is thought that the second and third columns of the orthogonal matrix U present the desired structure. Accordingly, the second and third columns of the orthogonal matrix U were selected as the desired structures. We refer to the structure of the second and third columns of the orthogonal matrix U a *hypothetical chromosome*. It then acquired the euchromatin region where the enhancer and promoter are predicted to be located. Moreover, the distance between the coordinates of the hypothetical chromosomes based on the Euclidean distance was determined.

(10) $$E_{i}=\sqrt{{\left(U_{i+1,1}-U_{i1}\right)}^{2}+{\left(U_{i+1,2}-U_{i2}\right)}^{2}}$$

The length of the DNA loop in the euchromatin region is only partially understood. In this study, we averaged $E_{i}$ every 50 kbp and used this value as the distance between the coordinates, the number of coordinates is reduced because it is averaged every 50 kbp. Accordingly, we included averages taken every 45, 40, and 35 kbp from both sides.

(11) $$E_{i}\mathrm{new}=\left\{\begin{array}{c} \frac{\Sigma_{j=1}^{k}E_{i+j}}{k}\;\;,i=0,k=5,6,\ldots ,9\;\\ \frac{\Sigma_{j=0}^{9}E_{i+j}}{10}\;\;,i=1,2,\ldots ,N-1\;\\ \frac{\Sigma_{j=0}^{k}E_{i+j}}{k+1}\;\;,i=N-9,k=8,7,\ldots ,4\; \end{array}\right.$$

**Figure 4:**
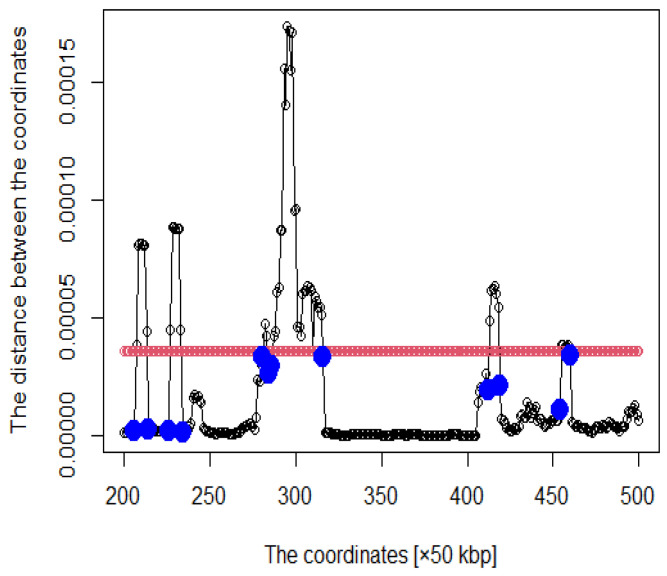
Distance plot between coordinates (The red line is the threshold and blue points are roots).

We proceeded to show the criteria for acquiring the coordinates as DNA loops. Five times the average distance between these genomic coordinates was set as the threshold. We focused on coordinates above the threshold value, which are considered to form DNA loops. However, because enhancers and promoters are at the ends of DNA loops, we also focused on coordinates below the threshold. In this study, the following criteria were established: $E_{0}$ was set as the threshold value and $E_{i}$ is the distance between coordinates. $n_{k}$ is the coordinate such that $E_{n_{k}}-E_{n_{k-1}}<0$, and $E_{n_{k+1}}-E_{n_{k}}>0$ is satisfied. Then, in arbitrary *i*, $n_{k}$ of $n_{k}<i<n_{k+1}$, where *i* satisfying $E_{i}>E_{0}$ is defined as $n_{k}^{\prime }$. Finally, we acquired $n_{k-1}^{\prime }$ to $n_{k+1}^{\prime }$ as a DNA loop. Thus, we considered that coordinates from the blue point to the next blue point in Figure 4 form a DNA loop.

### 2.4. Enrichment Analysis

BiomaRt was used to retrieve a list of genes from the obtained coordinates. The gene list obtained by BiomaRt was uploaded to g: Profiler [5] to identify functions, processes, and transcription factors related to enhancers and promoters. The coordinates acquired as euchromatin regions were subjected to enrichment analysis using g: Profiler. Enrichment analysis can reveal the functions of differentially expressed genes.

## 3. Results

### 3.1. Hypothetical Chromosomes

**Figure 5:**
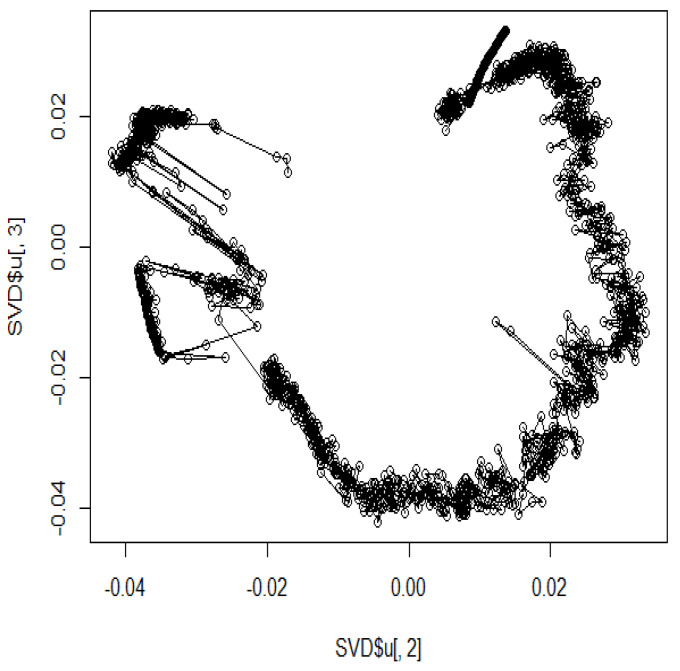
The hypothetical chromosomes 18 (0 bp–86,000 kbp).

The heat map of the Hi-C data after organizing these data by (1) is shown in Figure 3. Pairs with large values in the matrix indicate region pairs with a high contact probability. MDS was applied to the Hi-C data, and the resulting hypothetical chromosomes are shown in Figure 5. The euchromatin region was identified (Figure 4). The acquired euchromatin regions were summarized in Table S1 (Biological replicate 1) and Table S2 (Biological replicate 2).

### 3.2. Enrichment Analysis

We used BiomaRt [6] in R to retrieve genes from the obtained coordinates. Finally, the obtained euchromatin regions were subjected to enrichment analysis using g: Profiler [5]. The results are presented in Table 1 and Table 2. The functions and processes involved in transcription were also determined. The results of 0–360 min enrichment analysis were summarized in Table S3 (Biological replicate 1) and Table S4 (Biological replicate 2).

The DNA loops obtained from the reconstructed genome structure contained bioprocesses involved in transcription, such as the pre-transcriptional initiation complex and RNA polymerase II initiation complex, and transcription factors involved in cancer, such as CAMP responsive element binding protein 3 (CREB3) and forkhead box M1 (FOXM1). Estrogen receptor 1 (ER-alpha) is involved in regulating gene expression, and is associated with breast cancer [7]. FOXM1 plays an essential role in cell cycle progression; its expression peaks in the S and G2/M phases. FOXM1 upregulation occurs in most solid human cancers [8]. MAF BZIP transcription factor G (MafG) interacts with methionine adenosyltransferase a1 to regulate transcription; MafG is overexpressed in cancer cells [9]. RUNX family transcription factor 3 (AML2) forms a heterodimeric complex core-binding factor (CBF) with CBFB and functions as a tumor suppressor. The gene is frequently deleted or transcriptionally silenced in cancer cells [10]. Progesterone receptor (PR) is involved in regulating gene expression and is associated with breast cancer [7]. Retinoic acid receptor alpha (RARA) regulates transcription in a ligand-dependent manner; diseases associated with acute promyelocytic leukemia [11]. CREB3 encodes a transcription factor that is a member of the leucine zipper family of DNA-binding proteins. This protein binds to the CAMP-responsive element and regulates cell proliferation. The mRNA expression of CREB3 is higher in OS tissues than in normal tissues [12].

## 4. Discussion

### 4.1. Comparison with Previous Studies

**Figure 6:**
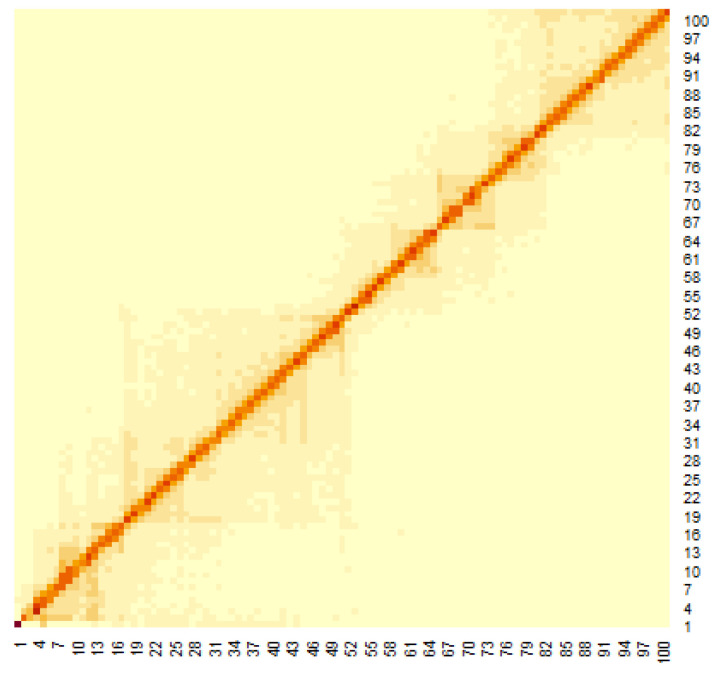
Heat map of 90 min Hi-C data of chromosomes 18 (0 bp–5000 kbp).

Previous studies have focused on identifying euchromatin regions or DNA loops in Hi-C maps [3]. The chromosome forms a topologically associating domain (TAD), and each TAD (A/B) compartment is organized with an average of 880 kb. The A compartment is loosely structured and serves as the transcriptionally active region, whereas compartment B is narrowly structured and serves as the transcriptionally inactive region. At the TAD level, loops and stripes/tracks were formed as sub-TADs (average size 185 kbp). Therefore, the TAD compartment is a rectangle that is approximately 880 × 880 kbp in size in the Hi-C map, and the A compartment is a rectangle that is approximately 185 × 185 kbp in the Hi-C map, where enhancers, promoters, and insulators form DNA loops. Figure 6 shows the existence of DNA loops of such sizes. As described above, previous studies identified these DNA loops. If only a rough TAD compartment must be identified, the method used in the previous study may be sufficient. However, detecting sub-TADs and DNA loops is markedly more complicated and will become more difficult as the resolution of Hi-C is improved.

**Figure 7:**
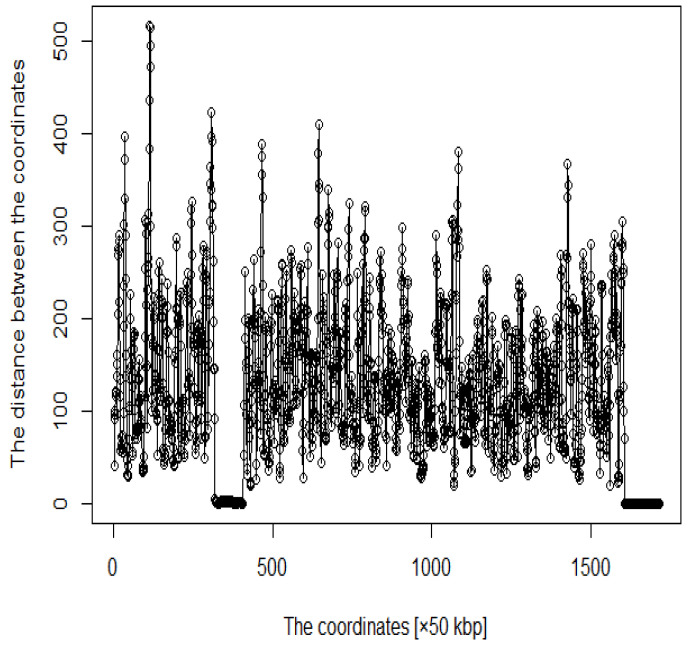
Distance plot between coordinates by StoHi-C.

In addition, several studies have used MDS to analyze Hi-C data for accurately reproducing 3D genome structures. For example, miniMDS [13] involves splitting high-resolution Hi-C data into several parts to which MDS is applied to low-resolution Hi-C data, and then the split high-resolution Hi-C data are reconstructed. The framework HiCRep evaluates the reproducibility of Hi-C data [14] using the stratum-coordinated correlation coefficient as a similarity measure to quantify differences between Hi-C contact matrices. Furthermore, it has been found that HiCRep/MDS method, which combines HiCRep with MDS, is robust to low per-cell sequence depths and that this robustness is further improved when high and low coverage cells are projected together [15]. Another framework for predicting 3D genomic structures using t-distributed stochastic neighbor embedding (t-SNE) is named as StoHi-C [16]. MDS has inherent problems with very sparse high-dimensional Hi-C datasets, whereas tSNE overcomes these limitations. This method can reproduce the characteristics of chromosome 3D structures more clearly than MDS in yeast Hi-C data, which are considered as suitable for recreating the 3D structure of chromosomes. The distances between the coordinates obtained from the 3D structure reproduced by the StoHi-C method are shown in Figure 7. As shown in Figure 7, attempts to precisely reproduce the 3D structure resulted in no significant difference in the distance between coordinates, even when acquiring DNA loops with a threshold value. Therefore, the enhancers and promoters cannot be precisely identified. We focused on the ones with a large number of Hi-C detections, although the distance between coordinates is large because the goal of this study was to identify enhancers and promoters. Therefore, we added weights as shown in Equation (1).

Based on our results, it is useful to obtain DNA loops by automatically visualizing the chromosome structure using MDS, as performed in this study.

### 4.2. Identification of Eigenvectors in MDS Representing the Actual Structure

We sought to identify the eigenvectors in MDS that represent the actual structure. The column of the orthogonal matrix U presenting the desired structure is unclear. However, in many cases, the second and third columns of the orthogonal matrix U are thought to present the considering structure. Herein, we performed a simple simulation, considering a circle with errors, such as $x_{i1}=\cos\left(2\pi \frac{i}{N}\right)+g\left(\lambda \right)$,$x_{i2}=\sin\left(2\pi \frac{i}{N}\right)+g\left(\lambda \right)$. Here, the function g is the error function and is defined as follows:

(12) g(λ)=λ,0≤λ≤0.5

**Figure 8:**
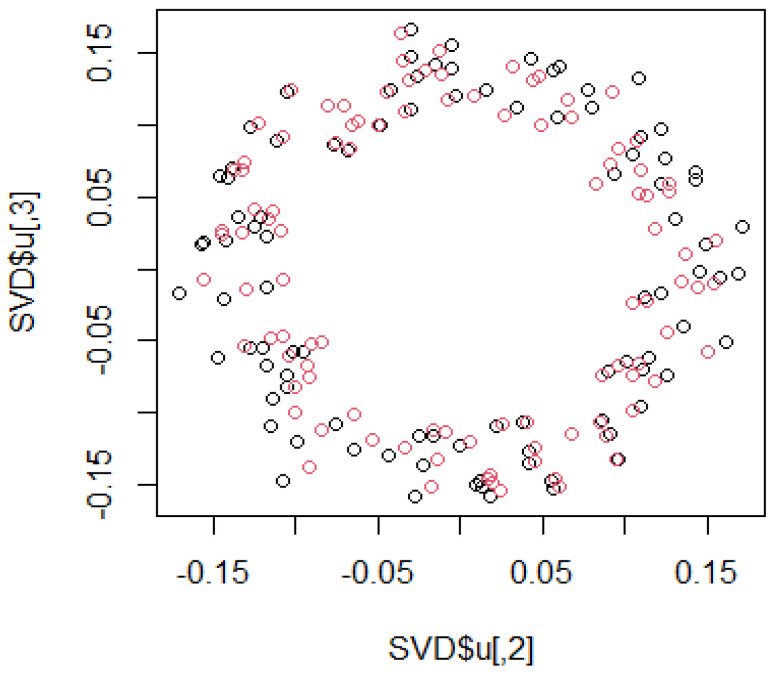
Simulation to identify the eigenvector with the original structure using sample data.

We used MDS to recover the original circle from the distance matrix; however, the second and third eigenvectors were found to represent the original circle. In such complex data with random numbers, the second and third eigenvectors typically represent the original structure (Figure 8).

## Figures and Tables

**Table 1:** Results of enrichment analysis of 90 min Hi-C data by g:Profiler.

Term_Name	Term_ID	Adjusted_*p*_Value
transcription preinitiation complex assembly	GO:0070897	$9.67\times 10^{-5}$
RNA polymerase II preinitiation complex assembly	GO:0051123	$5.90\times 10^{-3}$
immunoglobulin complex	GO:0019814	$6.72\times 10^{-49}$
immunoglobulin complex, circulating	GO:0042571	$3.97\times 10^{-8}$
DNA packaging complex	GO:0044815	$3.75\times 10^{-7}$
protein-DNA complex	GO:0032993	$5.36\times 10^{-4}$
transcription factor TFIID complex	GO:0005669	$9.22\times 10^{-3}$
RNA Polymerase I Promoter Opening	REAC:R-HSA-73728	$2.04\times 10^{-8}$
Transcriptional regulation by small RNAs	REAC:R-HSA-5578749	$5.98\times 10^{-7}$
Factor: ER-alpha; motif: TGACCYN	TF:M03547	$5.59\times 10^{-4}$
Factor: Foxm1; motif: NTGTTTRT	TF:M07255	$5.79\times 10^{-3}$
Factor: MafG; motif: CMATGACTCAGCAGA; match class: 1	TF:M07048_1	$1.04\times 10^{-2}$
Factor: AML2; motif: TGTGGTNNN	TF:M07372	$1.39\times 10^{-2}$
Factor: PR; motif: NNNNNNRGNACNNKNTGTTCTNNNNNN	TF:M00957_1	$2.66\times 10^{-2}$

**Table 2:** Results of enrichment analysis of 120 min Hi-C data by g:Profiler.

Term_Name	Term_ID	Adjusted_*p*_Value
transcription preinitiation complex assembly	GO:0070897	$2.93\times 10^{-4}$
RNA polymerase II preinitiation complex assembly	GO:0051123	$3.00\times 10^{-3}$
immunoglobulin complex	GO:0019814	$1.91\times 10^{-41}$
immunoglobulin complex, circulating	GO:0042571	$1.32\times 10^{-8}$
transcription factor TFIID complex	GO:0005669	$5.16\times 10^{-3}$
Factor: ER-alpha; motif: TGACCYN; match class: 1	TF:M03547_1	$1.15\times 10^{-4}$
Factor: RARA; motif: GAGGTCAAAAGGTCAAKK	TF:M08018	$2.84\times 10^{-3}$
Factor: AML2; motif: TGTGGTNNN	TF:M07372	$5.68\times 10^{-3}$
Factor: MafG; motif: CMATGACTCAGCAGA; match class: 1	TF:M07048_1	$8.21\times 10^{-3}$
Factor: CREB3; motif: NTGCCACGTCAYCN	TF:M04207	$4.57\times 10^{-2}$

## Data Availability

All data used in this study can be downloaded form GEO with GEO ID GSE141067.

## Supplementary Materials

The following are available online at https://www.mdpi.com/article/10.3390/genes12111671/s1, Table S1: Bio_rep1_DNAloop; Table S2: Bio_rep1_gProfiler_hsapiens; Table S3: Bio_rep2_DNAloop; Table S4: Bio_rep2_gProfiler_hsapiens.

## Appendix A

Listing A1 Sample code of the MDS simulation

> set.seed(1)
> theta <- seq(0,2∗pi,length=100)
> x <- cbind(cos(theta)+runif(100,0,0.5),sin(theta)+runif(100,0,0.5))
> plot(x)
> old <- svd(x)
> new <- data.matrix(dist(x))
> new <- svd(new^2)
> gc_old=c(mean(old$u[,1]),mean(old$u[,2]))
> gc_new=c(mean(new$u[,2]),mean(new$u[,3]))
> old_z=old$u[,1:2]-gc_old
> new_z=new$u[,2:3]-gc_new
> A=t(new_z)%∗%old_z
> rotsvd=svd(A)
> M=rotsvd$u%∗%rotsvd$v
> V=gc_new-M%∗%gc_old
> xy=cbind(old$u[,1]∗M[1,1]+old$u[,2]∗M[1,2]+V[1,],
+ old$u[,1]∗M[2,1]+old$u[,2]∗M[2,2]+V[2,])
> plot(new$u[,2],new$u[,3],xlab=’SVD$u[,2]’, ylab=’SVD$u[,3]’)
> points(xy[,1],xy[,2],col=2)
